# Reduced Attentional Control in Older Adults Leads to Deficits in Flexible Prioritization of Visual Working Memory

**DOI:** 10.3390/brainsci10080542

**Published:** 2020-08-11

**Authors:** Sarah E. Henderson, Holly A. Lockhart, Emily E. Davis, Stephen M. Emrich, Karen L. Campbell

**Affiliations:** Department of Psychology, Brock University, St Catharines, ON L2S 3A1, Canada; sh14jm@brocku.ca (S.E.H.); hl10ze@brocku.ca (H.A.L.); ed11zq@brocku.ca (E.E.D.); semrich@brocku.ca (S.M.E.)

**Keywords:** aging, working memory, visual working memory, attentional control

## Abstract

Visual working memory (VWM) resources have been shown to be flexibly distributed according to item priority. This flexible allocation of resources may depend on attentional control, an executive function known to decline with age. In this study, we sought to determine how age differences in attentional control affect VWM performance when attention is flexibly allocated amongst targets of varying priority. Participants performed a delayed-recall task wherein item priority was varied. Error was modelled using a three-component mixture model to probe different aspects of performance (precision, guess-rate, and non-target errors). The flexible resource model offered a good fit to the data from both age groups, but older adults showed consistently lower precision and higher guess rates. Importantly, when demands on flexible resource allocation were highest, older adults showed more non-target errors, often swapping in the item that had a higher priority at encoding. Taken together, these results suggest that the ability to flexibly allocate attention in VWM is largely maintained with age, but older adults are less precise overall and sometimes swap in salient, but no longer relevant, items possibly due to their lessened ability to inhibit previously attended information.

## 1. Introduction

The ability to maintain visual representations in working memory is critical in our dynamic environment, enabling the integration of events and information over time to facilitate decision-making and other higher-order executive processes. Unfortunately, working memory performance tends to decline with age [1,2,3], which may be attributed to age-related deficits in attentional control [4,5]. Recent models of visual working memory (VWM) suggest that attentional resources are flexibly allocated across stimuli according to their relative priority [6] rather than assigned to a set number of slots in working memory. Further, the ability to flexibly prioritize certain items in memory appears to rely on attentional control [7]. Thus, we might expect older adults to be less flexible in their allocation of attention, but this has yet to be explored in visual working memory.

### 1.1. VWM Resources Are Flexibly Allocated

The amount of information that can be held in working memory is limited [8]; as storage demands increase, either the fidelity of the representations in memory, or the number of maintained representations, decreases [9,10]. Two major theories are often used to explain the limits of working memory capacity: the discrete-capacity [9] and continuous-resource models [11]. The discrete-capacity model argues that there exist a fixed number of items that can be encoded with high fidelity in storage “slots”, with any items beyond that number failing to be encoded [12]. In opposition to this model is the continuous-resource model, which posits that working memory is not constrained by the number of representations that can be maintained, but rather by a limited pool of resources that can be distributed across items, such that lesser resource allocation per item results in lower-fidelity representations [11,13]. Thus, the continuous-resource model predicts that there is greater flexibility in the allocation of resources in working memory. However, most of this work has examined changes in performance by manipulating memory load, assuming equal division of resources between items, and largely ignoring the possibility that some items may be prioritized over others in accordance with one’s goals.

The flexible nature of working memory has been illustrated through work suggesting that the resources allocated to each item in working memory may not always be constant. Factors such as random fluctuations in attention at encoding [14], attentional cuing [12], rewards [15], and voluntary control (at least when load is low) [16] have all been shown to allow for between-item variability in quality of representation. Recent models of visual working memory have applied the continuous-resource model with the aim of assessing the flexibility of VWM resources more directly. Emrich and colleagues [6] used cues to manipulate the relative priority of different memoranda to examine whether spatial attention divided between items could account for error in VWM. Performance was found to be better predicted by the prioritization assigned to each item than by memory load alone. This relationship between attentional priority and memory performance followed a power law, suggesting that memory resources can be flexibly and continuously allocated to visual stimuli depending on attentional prioritization and, importantly, providing evidence that working memory performance can be improved through appropriate allocation of VWM resources [6,17].

### 1.2. Flexible Allocation Relies on Attentional Control

A flexible allocation of attention account of VWM is consistent with numerous models of VWM that identify attention as a critical factor in determining the information that is successfully encoded in working memory stores [18,19,20,21]. There are two distinct mechanisms through which attentional control may influence working memory encoding: through filtering/inhibiting distractors [22] and through biasing attention towards target items independent of filtering [6,15]. Filtering efficiency can be quantified using contralateral delay activity (CDA)—an event-related electrophysiological measure of sustained activity observed during the delay period of a memory task [9,23]. Increases in the number or complexity of items stored in VWM are associated with increased CDA amplitude, which saturates and appears to plateau when memory load reaches a few items [24]. However, low-capacity individuals show greater CDA amplitude than high-capacity individuals when distractors are present [25], reflecting storage of irrelevant items (i.e., poor filtering efficiency), and this relates to poorer target recall. Further, the association between poor attentional control and low VWM capacity has been linked to difficulty in overriding attentional capture, where low-capacity individuals have greater difficulty controlling spatial attention in the presence of distractors [26]. Thus, filtering efficiency appears to be a critical determinant of VWM performance [25] (although see [27]).

Similarly, the flexible prioritization of resources in working memory through attentional biasing can be tracked by a separate earlier electrophysiological component, the N2pc. Greater negativity of the N2pc is thought to reflect enhancement of task-relevant information prior to memory maintenance [28,29]. A larger N2pc has been observed for memory items prioritized by probabilistic cues with greater negative amplitude associated with higher-probability cues suggesting greater attentional enhancement of more probable items [7]. Thus, the flexible allocation of attention relies on both the suppression of distractors and the enhancement of task-relevant stimuli.

### 1.3. Aging, VWM, and Attentional Control

Consistent with attentional accounts of VWM performance, it has been established that VWM performance declines with age [3,30,31,32], and this is thought to be caused by concomitant declines in attentional control [4]. While older adults can sometimes use valid spatial cues to improve VWM performance in a manner similar to younger adults, they still display worse working memory overall [33]. This is particularly the case when distracting information is present. Past work suggests that older adults show impaired suppression of distracting information and are, thus, more likely to let that information into VWM, which in turn affects target recall [31,34,35]. Indeed, age-related increases in susceptibility to distraction have been linked to prolonged processing of distracting information [36]. This is the case even when distracting information is completely irrelevant to the task, as when auditory distractors were shown to have deleterious effects on visual working memory in older but not younger adults [37]. This work suggests that deficits in filtering efficiency or inhibition of irrelevant information can at least partially explain age differences in VWM [38,39,40] (although see [41]).

Recent work using mixture models to characterize VWM performance has provided further insight into the nature of age-related declines in VWM. These mixture models can identify the specific types of errors that participants make in a memory recall task, as well as define the precision or fidelity of visual representations in working memory. Using continuous response to measure memory for colour and orientation, Peich and colleagues [3] reported an age-related decline in VWM precision. They also showed an age-related increase in non-target errors or a greater tendency to report unprobed items from the original memory array, suggesting an age-related deficit in feature binding [42,43,44]. Similar findings were observed in a large, population-based sample who showed age-related declines in the fidelity and quantity of remembered items (coloured circles) [45]. Taken together, this work suggests that working memory representations become less precise with age, at least when attention is allocated equally across memoranda. It remains to be seen, however, whether older adults can flexibly allocate their attention when some items are prioritized over others in VWM.

### 1.4. Current Study

**Figure 1:**
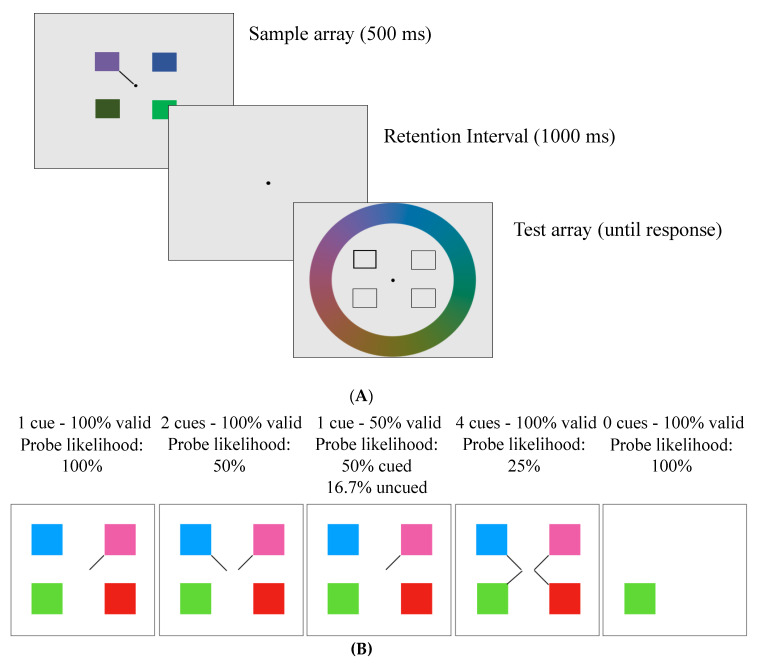
(**A**) Example trial from the delayed-recall task. Participants were shown a sample array for 500 ms with either one (in the 0 cue condition) or 4 squares (with 1, 2, or 4 line cues), which were blocked according to cue number and validity (conditions described in Table 1). Following a 1000 ms retention interval, a dark outline surrounded the square required to be recalled. Participants were required to select a colour on the colour wheel that matched the probed location in the sample array. (**B**) Sample arrays of all the trial conditions.

In this study, we aimed to examine whether older adults can flexibly allocate attentional resources in VWM in response to probabilistic cues. Using a delayed-recall paradigm, participants viewed coloured squares with probabilistic spatial cues that indicated the likelihood that each item would be probed (and thus, its priority), and then reported one of the squares using a continuous response (see Figure 1). The cues varied in number and validity across conditions such that the attentional resources to be allocated to each item would vary between 16.7% and 100% while maintaining a constant set size (except in a baseline single-item condition). We hypothesized that: (1) a flexible-allocation account would explain response error in younger adults [6] and that this pattern would largely hold for older adults; (2) older adults would show greater error overall reflecting a general decline in visual working memory (fidelity and capacity) [3]; and (3) older adults would have particular difficulty flexibly allocating attention when demands on attentional control are high (i.e., in the highest flexible attention prioritization condition) due to reduced attentional control [4,34,35].

## 2. Material and Methods

### 2.1. Participants

Participants included 20 older and 20 younger adults. One older adult was replaced because they scored less than 23 on the Montreal Cognitive Assessment (MoCA) [46,47] and two younger adults were replaced for not completing the second session. Older adults were recruited from a community participant pool and paid $10/h. Younger adults were recruited through a psychology student pool and received course credit for their time. Sample sizes were chosen in line with previous work from which the present study was adapted [6].

Older adults scored higher on the Shipley Vocabulary Test than younger adults, *t*(38) = 3.62, *p* < 0.001, in line with previous work [48]. There was no age difference in years of education (see Table 2 for all demographics). No participants reported being colour blind, and all participants met the criteria for normal colour vision after completing the Ishihara Test of Colour Vision.

### 2.2. Procedure and Stimuli

The study was approved by the Research Ethics Board at Brock University. The experiment was split into two, 1 h sessions (1.5 h for older adults) to reduce fatigue. All sessions took place in the afternoon with a maximum of seven days between sessions. All computer tasks were administered with PsychoPy (v.3.0.4) [49] on a 20 inch Dell CRT monitor (1024 × 768 px) with participants approximately 60 cm away from the monitor. In Session 1, after providing informed consent, participants were tested for colour blindness, and then completed three separate computer tasks: change detection (10 min), psychological distance triad (10 min) [50], and delayed recall (1/3 trials). In Session 2, participants completed the rest of the delayed-recall task (2/3 trials), followed by the MoCA, Shipley Vocabulary Test, and a demographics questionnaire (i.e., age, sex, education). Data from the change detection and psychological distance triad tasks are not reported here.

### 2.3. Delayed-Recall Task

This task was adapted from Emrich and colleagues [6]. Participants viewed four squares (1.8° × 1.8°) around a fixation dot (0.3° diameter) for 500 ms on a grey background. The squares were evenly spaced in four quadrants of the screen with 1, 2, or 4 line cues (see Figure 1). The line cues varied in their validity, and participants were explicitly told the validity of the cues at the start of each block. A total of four cued conditions were presented in separate blocks: 4 cues–100% valid (meaning one of those 4 squares would be tested); 2 cues–100% valid (meaning one of those 2 squares would be tested); 1 cue–50% valid (meaning there is a 50% chance that the cued square will be tested, and a 16.67% chance that each of the other squares will be tested); 1 cue–100% valid. There was also a no-distractor condition (0 cues–100% valid) in which participants viewed a single square that appeared randomly in one of the four quadrants with no cue (see Table 1). In all conditions except the 1 cue–50% valid condition, the uncued items would never be tested and thus could be ignored. For clarity, the probability of a cued item being probed has been termed “probe likelihood”.

Following a 1000 ms retention period, the test array appeared. The test array contained four square outlines in the same four locations as the sample array. One of the square outlines had a thicker border than the others indicating that participants had to recall the colour of the square that was in that location in the sample array. Participants selected the colours via a continuous-response colour wheel (14° radius) that appeared around the square outlines and contained all possible colours used in the experiment (see Figure 1). As participants moved a black probe around the colour wheel using the “c” (clockwise) and “m” (counterclockwise) keys, the tested square infilled with the colour of the wheel. When participants felt that the colour in the square was as close as possible to the correct colour, they pressed “space” to lock in their answer. Following participants’ decision, there was a 500 ms intertrial interval, then the next trial began. The no-distractor condition was presented in the same way, except that the test array had only one square outline in the same location as the sample array, which only included one square. Participants performed a total of 198 trials during Session 1 and 402 trials in Session 2. During each session, participants saw all five conditions in blocks (a breakdown of the number of trials per condition per day is provided in Table 1).

Sample colours were selected randomly from one of 360 unique colours obtained from a circular wheel on the CIE L*a*b* colour space with coordinates of a = −6 and b = 14 with a radius of 49, calibrated to the monitor, with a minimum distance of 30 degrees between sample colours.

### 2.4. Analysis

Responses in the delayed-recall task from Session 1 and Session 2 were merged using R. Due to a programming error, the 1 cue–50% valid condition only contained 100 trials, meaning that the uncued locations were only probed on 50 trials, providing fewer opportunities for participants to respond correctly to these trials than in the other conditions. To correct for this, these data were bootstrapped up to 200 trials (100 cued and 100 uncued) in R using the boot package [51,52]. Additionally, the 4 cue–100% trials accidentally included 100 extra trials, so only the first 100 were analysed in order to equate this condition to the other conditions. All reported analyses are based on the corrected data; however, it should be noted that the same pattern of results is observed when analyses are limited to the original (uncorrected) data (see Supplementary Figure S1 and Supplementary Table for uncorrected results).

#### 2.4.1. Response Error

**Power law vs. Linear fit.** Response error was calculated as the circular distance between the target value and the reported value on each trial, then the standard deviation of response error was calculated for each condition for each participant. The standard deviation of response error was chosen because it captures the precision of responses and is the statistic calculated by the mixture model [13]. A linear mixed-effects model was used to assess whether the relationship between probe likelihood and response error is better explained by a power law [6] relative to a simpler linear fit. This analysis was completed in R using the packages *lme4* [53], *lmerTest* [54], *car* [55], and *sjPlot* [56]. For the linear fit, response error was predicted by a fixed effect of probe likelihood with a random intercept for each participant. This is analogous to a repeated-measures ANOVA, which allows each participant to vary from each other, but the effect of the predictor (probe likelihood) is the same for each participant. Because a power-law function is linear when both the predictor (x) and outcome (y) are log transformed, the log of SD response error was predicted by a fixed effect of the log of probe likelihood with a random intercept for each participant. Degrees of freedom were estimated using the Kenward–Roger approximation, which is known to reduce bias in small-sample parameter estimation in linear models [57]. Model fit is reflected by AIC values (with lower values indicating better fit) and these were compared between models. After finding the best fitting model for each group, the marginal R-squared values were compared between groups using Fisher’s Z test for independent sample comparison.

**Key comparisons.** The hypothesis that attentional resources are flexibly allocated across memoranda leads to two specific predictions. First, items of equivalent probe likelihood (i.e., similar priority) should receive similar amounts of attention and, thus, have similar response error. Specifically, cued items from the 1 cue–50% valid condition should receive the same amount of attention as 2 items in the 2 cues–100% valid condition. To test the hypothesis of no difference between these conditions, response error was submitted to a Bayesian repeated-measures ANOVA with condition (1 cue–50% valid vs. 2 cues–100% valid) as a within-subjects factor and age group (young vs. old) as a between-subjects factor. The second key prediction is that items with different probe likelihood, but which are similarly cued (i.e., different cue validity and thus different priority), will not receive similar amounts of attention and should show differences in response error. To test this hypothesis, response error was submitted to a Bayesian repeated-measures ANOVA with cue validity condition (1 cue–100% valid vs. 1 cue–50% valid) as a within-subjects factor and age group (young vs. old) as a between-subjects factor.

For ease of reading, the BF_01_ is reported when the prediction was in favour of the null hypothesis and BF_10_ when the prediction was against the null hypothesis. These analyses were run in JASP [58]. For reference, a BF_01_ > 3 (equivalent of BF_10_ < 0.33) suggests moderate evidence for the null hypothesis and BF_10_ > 3 suggests moderate evidence for the alternative hypothesis; values greater than 10 are considered strong evidence in the respective directions [59]. Additionally, BF_incl_ refers to the posterior probability that the inclusion of the model term or interaction would produce a model that explains the observed data. Models that include the interaction term always include the lower-order terms, so where inclusion of the interaction term is justified, only the interaction BF_incl_ is reported. Evidence for the exclusion of the condition term (BF_incl_ < 0.33) suggests no difference between conditions and in these cases, the best supported model does not include the condition term.

**Spatial Cue Utilization.** Although not a prediction of the flexible-allocation account of memory and attention resources, we included a no-distractor condition that could verify whether any observed age differences were due to a catastrophic failure to use the spatial cues. The 0-cue and 1-cue–100% conditions both require a single item to be remembered, but the 1 cue–100% condition also includes 3 distracting items that should be ignored. By comparing the 1 cue–100% valid to the no-distractor condition, we can determine whether both groups are able to use spatial cues to effectively filter distractors under low flexible-allocation demands. Response error from these conditions was submitted to a Bayesian repeated-measures ANOVA with condition (1 cue–100% valid vs. no-distractor) as a within-subjects factor and age group (young vs. old) as a between-subjects factor. Performance should be high and equivalent in both conditions if participants are able to use a highly predictive cue to direct attention.

#### 2.4.2. Mixture Model

Response error in the delayed-recall task was split by condition and modelled using the three-component mixture model [13] in MemToolbox for MATLAB R2017a [60]. This mixture model gives output parameters for guess rate (proportion of random responses), non-target rate (proportion of responses centred around one of the non-target items), and response precision (the standard deviation of the circular von Mises distribution, centred around the target value) in degrees. The approximate number of trials used to estimate the precision parameter (correct trials) can be calculated according to the following formula: trials = (total trials) × (1-guess rate + non-target rate). Since precision is based on the circular normal (von Mises) distribution of response error after accounting for a uniform distribution containing guesses and non-target responses, this method means precision is calculated on correct trials only, but because of the continuous-response method, correct/incorrect is not a binary decision and is independent for each participant. If there were under 10 trials used to calculate a participant’s precision in any condition, the modelled precision output for that condition was removed from the analysis. This criterion was determined so that the precision parameter would be a reliable estimate while retaining as many older participants as possible. This criterion resulted in the removal of the uncued response data from the 1 cue–50% valid condition in 1 young adult and 5 older adults. After excluding these participants, the average number of correct trials in young adults was 60.1 with a mean precision of 26.6 for this condition, while older adults had an average of 40.9 correct trials with a mean precision of 31.6.

Parameter estimates (guess rate, non-target rate, and precision) were submitted to separate Bayesian repeated-measures ANOVAs with probe likelihood (100%, 50%, 25%, 16.67%) as a within-subjects factor and age group (young vs. old) as a between-subjects factor. Models for each factor and the interactions were compared to a null model. These analyses were run in JASP [58].

## 3. Results

### 3.1. Response Error

We first examined raw response error to compare whether older and younger adults could flexibly allocate VWM resources in a similar way. Previous work with younger adults has shown that response error is affected by probe likelihood in a continuous fashion according to a power law [6,7,17]; thus, our first question was whether older adults’ response error could similarly be characterized by a power law. To this end, a linear mixed-effect model was run on the log-transformed response error data with log-transformed probe likelihood as a predictor, and this was compared to a simpler linear fit (i.e., untransformed variables). The analysis was first run on the whole sample with group as a predictor and then run on each group independently. The whole-group analysis revealed a significant group difference such that older adults had greater error overall, (*t*(42.11) = 5.40, *p* < 0.001). The power law offered a better fit to the data than the linear fit (AIC_power law_ = −11.11 vs. AIC_lineaar_ = 7450.42) in the sample as a whole and also in the younger (AIC_power law_ = 5.63 vs. AIC_linear_ = 3511.97) and older groups alone (AIC_power law_ = 7.29 vs. AIC_linear_ = 3857.24). To test whether the power law offered a better fit to the data in either group, a Fisher’s Z test compared the marginal R-squared values between groups. While the fit was numerically better in young adults, the difference was not significant (R^2^_Older Adults_ = 0.388, R^2^_Young Adults_ = 0.429, *Z* = 0.158, *p* = 0.437). Thus, both younger and older adults appear to use the attentional cues to flexibly allocate VWM resources.

#### 3.1.1. Key Comparisons

Supporting a flexible-allocation account, both groups reported items of equal priority with equal error (i.e., 1 cue–50% valid vs. 2 cues–100% valid; see Supplementary Figure S1). A Bayesian repeated-measures ANOVA only found support for the inclusion of group (BF_incl_ = 695.69) and moderate support for the exclusion of condition (1 cue–50% valid vs. 2 cues–100% valid; BF_incl_ = 0.32), with no support either way for the interaction term (BF_incl_ = 0.77); thus, there was strong evidence that the best model was a group-only model (Model BF_10_ = 868.85). Analysis of the group difference showed strong evidence that older adults reported more error overall (BF_10_ > 1000). In other words, although older adults were worse overall, they showed a similar ability to flexibly allocate their attention according to the cue validity.

Further, both groups seem to differentiate a single-cued item by cue validity (i.e., 1 cue–100% valid vs. 1 cue–50% valid; see Supplementary Figure S1). A Bayesian repeated-measures ANOVA found strong evidence for the inclusion of the interaction of age group and cue validity (interaction BF_incl_ = 25.20); there was strong support for a model that included group, cue validity, and their interaction (Model BF_10_ > 1000). Analysis of the group difference showed there was strong evidence that older adults report greater error overall (BF_10_ > 1000), and that there was greater error in the 1 cue–50% valid condition than in the 100% valid condition (BF_10_ >1000). To follow-up the interaction, Bayesian independent samples t-tests showed a larger group difference in the 50% valid (BF_10_ = 319.37) than the 100% valid (BF_10_ = 109.74) single-cue condition. Taken together, these key comparison results suggest that participants were using cue validity to appropriately allocate memory and attentional resources.

#### 3.1.2. Spatial Cue Utilization

**Figure 2:**
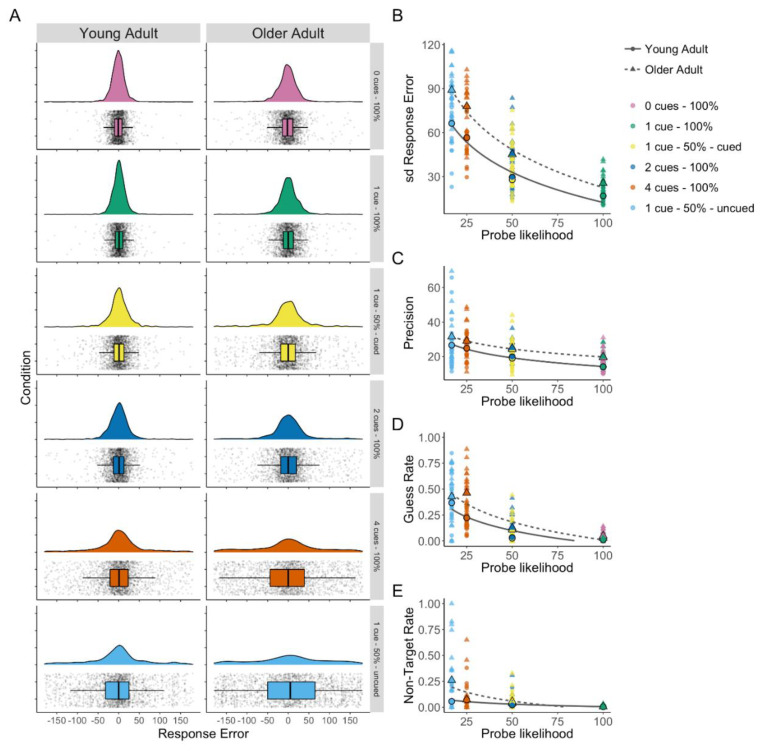
(**A**) Density plots of response error (distance from the target colour in degrees) by condition with box plots and all data points shown for older and young adults (based on raincloud-plots [61]). (**B**) Response error (SD) by probe likelihood. (**C**) Mixture model precision (error in degrees from probed colour) by probe likelihood. (**D**) Mixture model guess rate by probe likelihood. (**E**) Mixture model non-target response rate by probe likelihood.

Although the previous analyses suggest both groups were able to use spatial cues to allocate attention and memory resources, we further examined older adults’ ability to use spatial cues by comparing the 0-cue (no-distractor) condition to the 1 cue–100% valid condition. The no-distractor and 1 cue–100% both require a single item to be remembered, but the 1 cue–100% condition also includes 3 distracting items that should be ignored. Distributions of the response errors for each condition by older and young adults can be seen in Figure 2A. A Bayesian repeated-measures ANOVA found best support for a group-only model (Model BF_10_ = 438.36). There was moderate support for no effect of condition, given the evidence for exclusion of condition term (BF_incl_ = 0.32). Additionally, there was no support either way for the inclusion of the interaction between age group and condition (BF_incl_ = 0.42). Follow-up Bayesian paired sample t-tests within each group found moderate evidence of no difference between conditions (Older Adults, BF_01_ = 8.414; Young Adults, BF_01_ = 4.341). This suggests that both groups were able to effectively use the spatial cues to direct attention and ignore distractors when a single item was cued with 100% validity. While analysis of the group difference again showed strong evidence that older adults had higher error overall (BF_10_ > 1000).

### 3.2. Mixture Model

The three-component mixture model enables the assessment of age-related changes in separate aspects of working memory performance using parameter estimates of guess rate, non-target error rate, and precision. Flexible allocation of attention predicts that precision will decrease as attention allocated to the probed stimuli decreases [6]. Further, older adults were expected to have lower precision overall [3]. A Bayesian repeated-measures ANOVA found the best support for a model including probe likelihood and group (both BF_incl_ > 6) but excluding the interaction term (BF_incl_ = 0.11), Model BF_10_ > 1000. Analysis of the group difference showed strong evidence that older adults were less precise (BF_10_ = 187.89; see Figure 2C), and this was consistent across the probe likelihood conditions.

Guess rate was expected to increase as probe likelihood decreased, reflecting poorer memory for less attended items across both age groups. Additionally, older adults were expected to show higher guess rates overall, reflecting lower visual working memory abilities. A Bayesian repeated-measures ANOVA found the strongest support for the model including a probe likelihood × group interaction (BF_incl_ = 54.09), Model BF_10_ > 1000 (see Figure 2D). Analysis of the group difference showed strong evidence that older adults generally had higher guess rates (BF_10_ = 19.44). To investigate the interaction, Bayesian independent sample t-tests were performed. There was no evidence of a group difference at either the highest probe likelihood (1 cue–100% valid) or the lowest probe likelihood (1 cue–50% uncued); however, there was a pattern of increasingly larger differences between groups as probe likelihood decreased from the 0 cue–100% valid condition (BF_10_ = 3.61) to 1 cue–50% valid cued (BF_10_ = 5.92), 2 cues–100% valid (BF_10_ = 15.64), and as probe likelihood further decreased there is strongest evidence for a difference in 4 cues–100% valid (BF_10_ = 139.98).

Finally, non-target error rate (or “swap rate”) was expected to increase in older but not younger adults when flexible prioritization demands increased, but memory load remained constant.

A Bayesian repeated-measures ANOVA found the strongest support for the model including a probe likelihood × group interaction (interaction BF_incl_ = 126.884), Model BF_10_ > 1000 (see Figure 2E). To investigate the interaction, Bayesian independent sample t-tests were performed. Only the 1 cue–50% uncued condition showed moderate evidence for a difference between age groups (BF_10_ = 4.03), all other conditions showed inconclusive evidence (BF_10_s < 0.48) except the 1 cue–100% valid condition (BF_01_ = 3.02) and the 4 cue–100% condition (BF_01_ = 3.235), for which there was moderate evidence of no difference.

**Figure 3:**
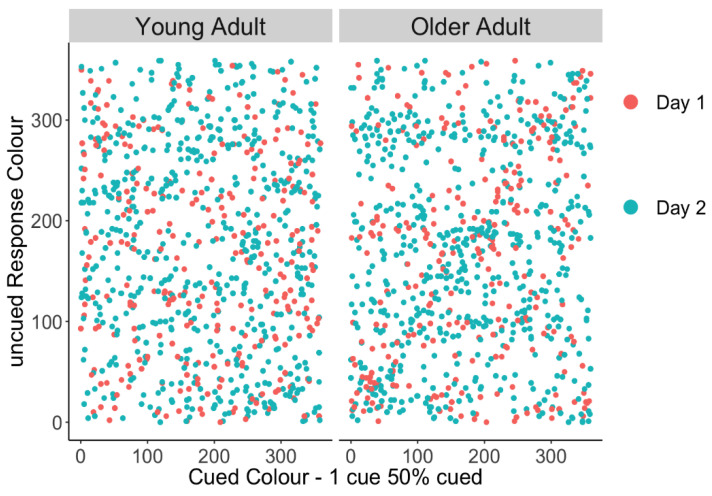
Uncued responses made towards the cued colour for young and older adults. Erroneous responses to the cued item should fall on the diagonal, and a linear trend would suggest a tendency to report the cued item in place of the uncued probe, as is apparent in the older group.

We tested whether older adults’ higher non-target error rate on 1 cue–50% uncued trials was due to them swapping in the cued item or whether were they equally likely to report the other uncued items. This potential bias towards the cued item can be graphically depicted in a scatter plot of responses by having the cued colour on the x-axis and the reported colour on the y-axis, where responses to the cued item colour should fall on the diagonal. Thus, a linear trend would suggest a tendency to report the cued item in place of the uncued probe, as is apparent in older adults’ responses in Figure 3. To quantify this relationship, a linear mixed-effect model was performed separately in young and older adults predicting the response colour from the target (probed, lower priority) colour, and secondly the cued (unprobed, higher priority) colour as fixed effects (random intercepts for participants). Target colour should be predictive of response colour if participants are responding correctly; however, in this case, we were primarily interested in whether the remaining error could be explained by the high-priority cued item. Table 3 shows that in young adults, the target colour is a significant predictor of response colour, but the cued colour is not. In contrast, older adults’ responses were predicted by both the target colour and the cued colour, suggesting that they often reported the unprobed, higher-priority item when they were probed to respond with one of the low-priority items.

## 4. Discussion

The goal of the present study was to determine how age differences in attentional control affect VWM performance when attention is flexibly allocated amongst targets of varying priority. Replicating previous work with younger adults, we show that item priority predicts VWM performance according to a power law, and we extend this finding to older adults. This suggests that despite age-related declines in attentional control, both younger and older adults can flexibly allocate attentional resources according to item priority. Further evidence of this can be taken from our key comparisons: both groups showed equivalent error for items with equivalent probe likelihood and differences in error when the number of cues was the same but probe likelihood differed, suggesting that attention was allocated to items based on priority. A three-part mixture model was used to assess distinct aspects of working memory, revealing that older adults had overall lower precision and higher guess rates. Critically, older adults made more non-target errors when demands on attentional control were highest (i.e., when uncued items in the 1 cue–50% valid condition were tested), and these errors were often the result of swapping in the cued item. This may be because older adults had difficulty inhibiting the more salient (50% likely) item when it was no longer relevant at retrieval (e.g., [62]).

We replicate previous findings in younger adults by showing that the proportion of resources allocated to an item is a useful predictor of VWM performance. A power law where response error was predicted by probe likelihood in a continuous fashion was found to fit the raw error of VWM performance in younger adults and offered a better fit than a simpler linear model. Thus, our data support the long-standing proposal that top–down attentional control and VWM performance are closely intertwined [63,64] and fit with recent models of VWM as a continuous and flexible resource relying on the allocation of spatial attention [6,7].

Importantly, we sought to determine whether this flexible allocation of attention account of working memory performance could similarly explain older adults’ response error data. We found that older adults’ VWM performance was well fit by a power law where probe likelihood predicts performance. In further support of this model, both older and younger adults reported items of equal priority with equal error (1 cue–50% valid vs. 2 cues–100% valid), and differentiated a single-cued item by its validity (1 cue–100% valid vs. 1 cue–50% valid), suggesting that item priority, rather than the number of cues, determined the allocation of attentional resources. Finally, we also examined older adults’ ability to use spatial cues to shift their attention and ignore irrelevant distractors. We compared a no-distractor condition (one item only) to the 1 cue–100% condition, which includes additional distractor items that should be ignored. Previous work has shown that older adults show a greater distractor-related deficit in working memory performance when memory load is high, thus, we may expect both groups to show equivalent performance across conditions wherein the memory load is one item [65]. Indeed, both groups showed no difference between these conditions, suggesting that older adults can use highly predictive cues to direct spatial attention and improve VWM performance [33,66,67,68]. Taken together, these results suggest that similar to younger adults, older adults allocate attention in a flexible manner according to item priority (albeit with less accuracy), which influences what information is encoded in short-term memory.

Previous work has applied a three-part mixture model of working memory to investigate older adults’ VWM in terms of guess rate, precision, and non-target guess rate revealing that age differentially affects these parameters [3,33]. We therefore applied this method to allow for more specific assessments of age-related differences in VWM performance at different attentional priority levels. In terms of precision, we found that older adults have lower precision than younger adults overall and that the fidelity of visual representations in working memory was similarly affected by probe likelihood in both groups [6]. This is in line with previous work showing an age-related decline in the precision of VWM [3].

For guess rate, lower-priority items had a higher guess rate and older adults made more guesses overall, likely reflecting an age-related decrease in working memory resources [30]. Further, the effect of decreasing priority had a steeper effect on older adults’ guess rate, who guessed more than younger adults particularly at lower levels of priority. There were no age differences observed in guess rate for the lowest priority condition (1 cue–50% uncued), however, this reflected an increased proportion of non-target errors made by older adults whose cumulative error rate remained significantly higher (Supplemental Figure S2). This likely reflects that as fewer resources are allocated to an item, older adults reach the limit of their available resources [69] and, thus, fail to adequately encode and/or recall some items of lower priority.

Non-target guess rate or swap rate was expected to increase with age, particularly when flexible prioritization demands were high, and we found evidence to this effect. Older adults made more swap errors only when they needed to report an uncued item in the 1 cue–50% condition (i.e., when another item was more highly prioritized). This appeared to be due to older, but not younger, adults reporting the (higher-priority) cued item when the (lower-priority) uncued item was probed. Older adults’ tendency to report the salient, but no longer relevant, cued item may reflect their lessened ability to inhibit previously attended information when it becomes task irrelevant, a process that is sometimes referred to as deletion or working memory updating [39,40,62,70,71]. In line with this proposal, older adults have recently been shown to display greater neural activation for no-longer-relevant items during working memory maintenance, an effect that predicts worse recall performance for relevant items [72]. Thus, older adults may have difficulty inhibiting irrelevant items especially when they were once assigned higher priority.

Alternatively, higher reporting of the cued item could reflect a difference in strategy between the groups, such that older adults may have limited their attention to the higher-priority cued item and ignored the lower-priority uncued items (i.e., giving 100% of their attention to the 50% likely item). Refuting this possibility, older adults showed equivalent performance in the 1 cue–50% and 2 cue–100% conditions, when cued items should have received 50% of attentional resources (suggesting that they only gave 50% of their attention to the cued item in the 1 cue–50% condition). Further, if older adults were focusing all their attention on the cued item in the 50% valid condition, we would instead expect performance in this condition to be similar to the other single-cue condition (1 cue–100% valid condition), but error was markedly lower in the 1 cue–100% condition (see Supplementary Figure S1, lower panel). Thus, the swap errors observed in older adults are more likely a product of poor inhibitory control, rather than an ill-adapted strategy.

This work has implications for other populations with poor attentional control, such as children [73] and those diagnosed with ADHD [74], depression [75], and anxiety [76]. For example, deficits in attentional control in ADHD have been linked to deficits in working memory [74]. Recent work investigating VWM in those with ADHD has revealed that performance deficits relate to reduced N2pc amplitude, an EEG component known to be associated with flexible allocation of attention through attentional control [7]. However, these studies have largely focused on memory load and thus, the potential role of attentional control in flexibly allocating attention has been left largely unexplored. Future work may benefit from applying this flexible-attention account of VWM and using mixture models to elucidate the mechanisms through which poor attentional control may influence VWM.

In conclusion, we show that similar to younger adults, older adults’ VWM performance relies on the flexible allocation of attention. Older adults show lower precision overall, potentially reflecting lower fidelity of visual representations in working memory and higher guess rates. When demands on flexible resource allocation were highest (on 1 cue–50% uncued item trials), older adults often reported the higher priority (but no longer relevant) cued item, possibly reflecting an age-related inhibitory deficit. The current data elucidates the relationship between flexible allocation of attention models of visual working memory, and the age-related inhibitory deficit by showing that the deletion of no-longer-relevant information is impaired in older adults when attention must be flexibly divided amongst items and task demands are high. Future work should incorporate additional flexible prioritization conditions to further disentangle the relationship between load and probe likelihood and assess potential differences in strategy use between older and younger adults.

## Figures and Tables

**Table 1:** Condition parameters and trial information.

Condition	Number of Items	Number of Cues	Memory Load	Cue Validity (%)	Probability of Cued Item Probed (%)	Probability of Uncued Item Probed (%)	Number of Trials Day 1	Number of Trials Day 2
0 cues–100%	1	0	1	--	--	--	33	67
1 cue–100%	4	1	1	100	100	0	33	67
1 cue–50%	4	1	4	50	50	16.67	33	67
2 cues–100%	4	2	2	100	50	0	33	67
4 cues–100%	4	4	4	100	25	0	66	134

**Table 2:** Demographics.

Group	Age (Years)			Education (Years)			Vocabulary			MoCA		
	M	Range	SD	M	Range	SD	M	Range	SD	M	Range	SD
Younger	21.10	18–26	2.34	15.65	12–20	1.87	0.74	0.60–0.98	0.12	-	-	-
Older	73.32	65–85	4.92	15.25	9–40	6.38	0.87	0.45–1	0.12	25.75	23–29	1.71

MoCA: Montreal Cognitive Assessment. Vocabulary is reported as proportion correct. Age for one older adult participant was missing.

**Table 3:** Linear mixed-effects model.

Predictors	Young Adult				Older Adult			
	*B*	CI	*p*	df	*B*	CI	*p*	df
(Intercept)	91.09	77.78–104.40	<0.001	126.16	98.67	85.09–112.25	<0.001	205.80
Target Colour	0.45	0.42–0.49	<0.001	1996.40	0.22	0.17–0.26	<0.001	1996.72
Cued Colour	0.00	−0.04–0.04	0.882	1991.04	0.17	0.13–0.21	<0.001	1996.82
Marginal R^2^ Conditional R^2^	0.233 0.258				0.067 0.084			

## Supplementary Materials

The following are available online at https://www.mdpi.com/2076-3425/10/8/542/s1, Figure S1: Key comparison of error for conditions with equal priority and equal cue number, Figure S2: Combined error rate calculated by adding guess rate and non-target error rate for each age group and probe likelihood, Figure S3: Results figure with uncorrected data, Table S1: Summarizing the differences in results with uncorrected data.
