# Supplementary material for: Reduced Attentional Control in Older Adults Leads to Deficits in Flexible Prioritization of Visual Working Memory
Source: Brain Sci. 2020 Aug 11;10(8):542. doi: 10.3390/brainsci10080542 (PMC7466080; doi:10.3390/brainsci10080542)
Supplement: Supplementary file 1 [file brainsci-10-00542-s001.pdf]

Supplementary Material

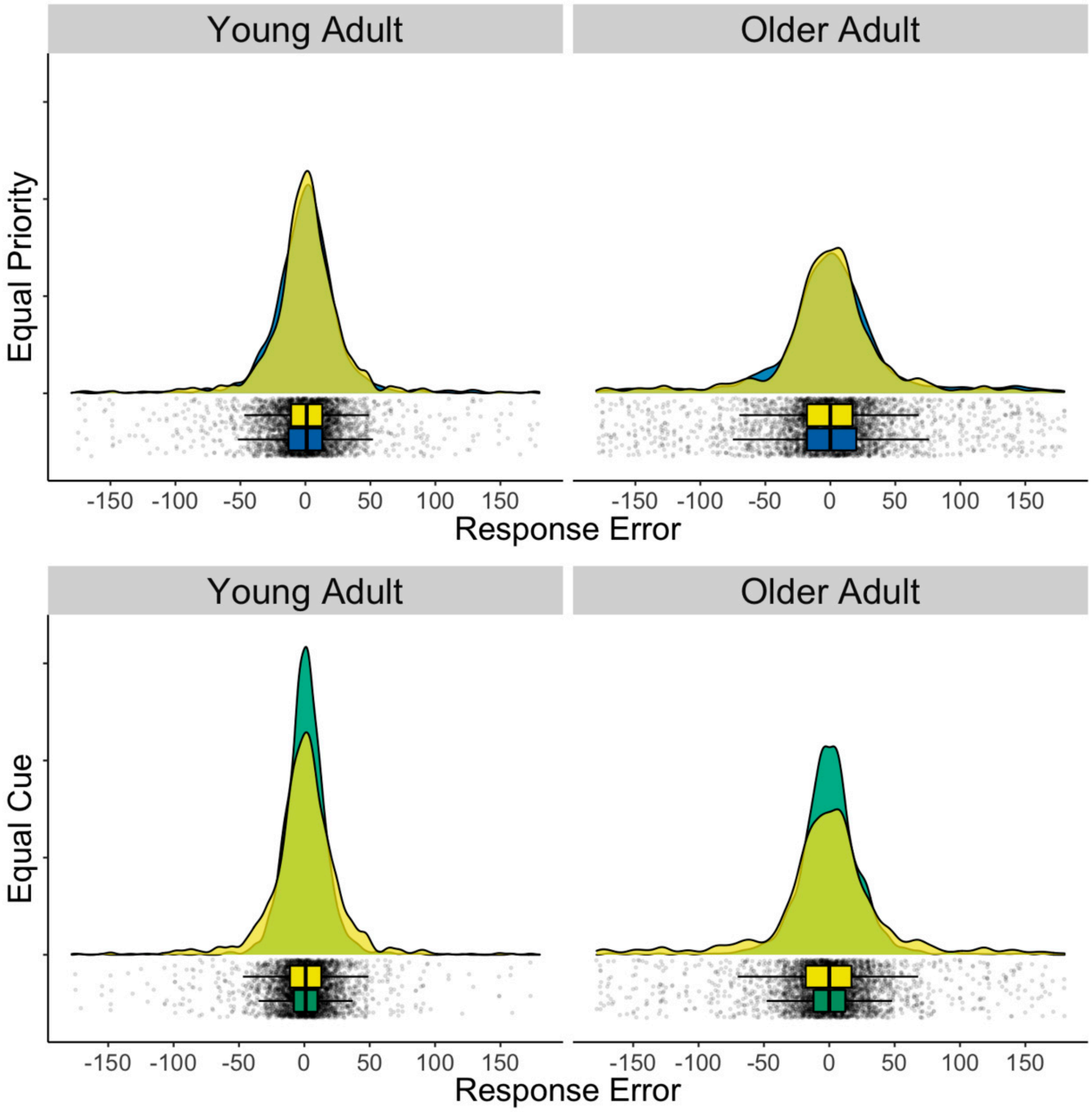

Figure S1. Top panel: Key comparison of error for conditions with equal priority (yellow = 1 cue – 50% valid; blue = 2 cues – 100% valid) reflecting similar performance at equal priority in young and older adults. Bottom panel: Key comparison of error for equal cue number (green = 1 cue – 100% valid; yellow = 1 cue – 50% valid) reflecting differentiation of cues based on priority when the number of cues was the same.

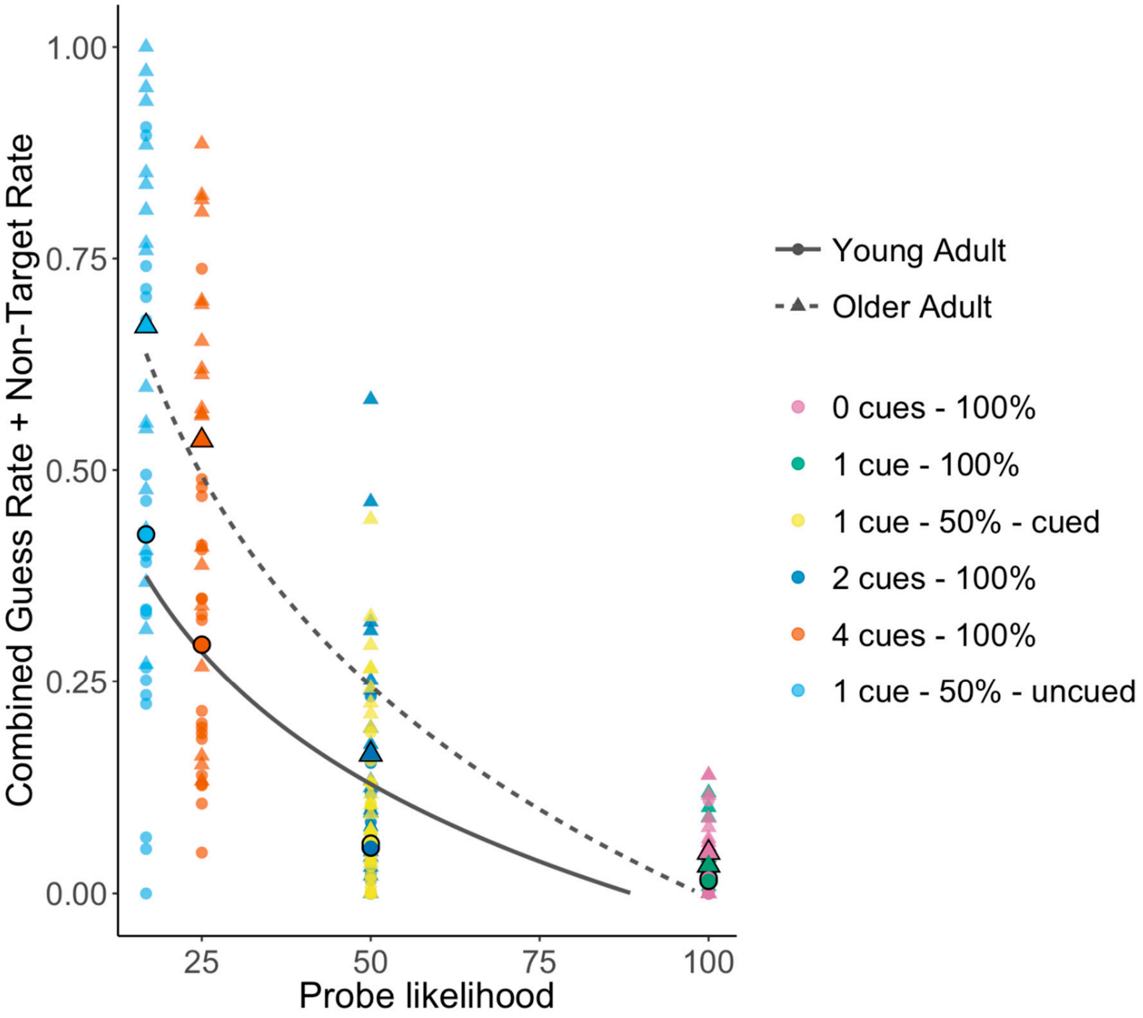

Figure S2. Combined error rate calculated by adding guess rate and non-target error rate for each age group and probe likelihood.

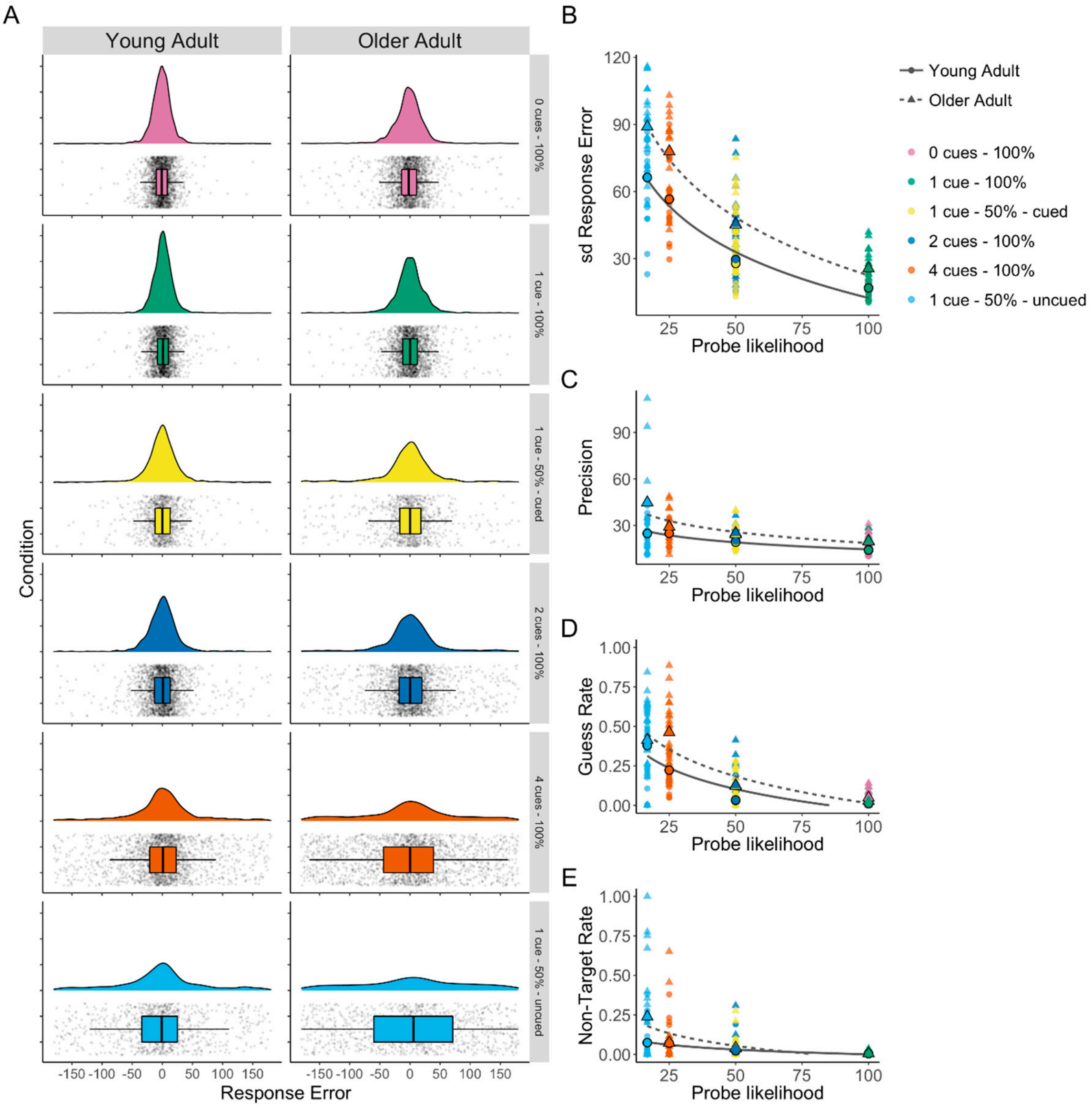

Figure S3. Results figure with uncorrected data.

Table S1. Summarizing the differences in results with uncorrected data.

Model Parameter	Statistic		Corrected	Uncorrected
Precision	Model BF:	Without Interaction	1.432e +13	1.686e +9
		With Interaction	4.499e +11	6.032e +9
		Interaction BF incl.	0.114	13.590
	t-test BF_{10}	0 cues 100%		141.962
		1 cue 100%		6069.968
		2 cue 100%		71.140
		1 cue 50% - cued	2.672	6.052
		4 cues 100%		0.869
		1 cue 50% - uncued	0.469	2.845
Guess Rate	Model BF	With Interaction	2.564e +39	6.988e +42
		Interaction BF incl.	54.086	216.344
	t-test BF_{10}	1 cue 50% - cued	5.917	25.299
		1 cue 50% - uncued	0.388	0.338
Non-target Rate	Model BF	With Interaction	4.130e +5	6.092e +4
		Interaction BF incl.	126.884	5.569
		Group BF incl.	43.664	2.115
	t-test BF_{10}	1 cue 50% - cued	0.483	0.388
		1 cue 50% - uncued	4.034	1.803
